# Supplementary material for: MetaQTL: a package of new computational methods for the meta-analysis of QTL mapping experiments
Source: BMC Bioinformatics. 2007 Feb 8;8:49. doi: 10.1186/1471-2105-8-49 (PMC1808479; doi:10.1186/1471-2105-8-49)
Supplement: Additional File 7 — MetaQTL Package : jar file and tutorial. This Zip archive contains both the MetaQTL JAR file and the files of the tutorial. [file 1471-2105-8-49-S7.zip › org.inra.metaqtl/doc/org/metaqtl/main/CmdLineParser.Option.html]

CmdLineParser.Option


|  |  |  |
| --- | --- | --- |
| **Overview** \| **Package** \| **Class** \| **Use** \| **Tree** \| **Deprecated** \| **Index** \| **Help** | |  |
| **PREV CLASS**   **NEXT CLASS** | **FRAMES**    **NO FRAMES**     **All Classes** |  |
| SUMMARY: NESTED \| FIELD \| CONSTR \| METHOD | DETAIL: FIELD \| CONSTR \| METHOD |  |


---


## org.metaqtl.main Class CmdLineParser.Option

```
java.lang.Object
  org.metaqtl.main.CmdLineParser.Option
```

**Direct Known Subclasses:**: CmdLineParser.Option.BooleanOption, CmdLineParser.Option.DoubleOption, CmdLineParser.Option.IntegerOption, CmdLineParser.Option.LongOption, CmdLineParser.Option.StringOption

**Enclosing class:**: CmdLineParser

---

``` public abstract static class CmdLineParser.Option extends java.lang.Object ```

Representation of a command-line option

---

| **Nested Class Summary** | |
| --- | --- |
| `static class` | `CmdLineParser.Option.BooleanOption` |
| `static class` | `CmdLineParser.Option.DoubleOption`             An option that expects a floating-point value |
| `static class` | `CmdLineParser.Option.IntegerOption`             An option that expects an integer value |
| `static class` | `CmdLineParser.Option.LongOption`             An option that expects a long integer value |
| `static class` | `CmdLineParser.Option.StringOption`             An option that expects a string value |


| **Constructor Summary** | |
| --- | --- |
| `protected` | `CmdLineParser.Option(char shortForm, java.lang.String longForm, boolean wantsValue)` |
| `protected` | `CmdLineParser.Option(java.lang.String longForm, boolean wantsValue)` |


| **Method Summary** | |
| --- | --- |
| `java.lang.Object` | `getValue(java.lang.String arg, java.util.Locale locale)` |
| `java.lang.String` | `longForm()` |
| `protected  java.lang.Object` | `parseValue(java.lang.String arg, java.util.Locale locale)`             Override to extract and convert an option value passed on the command-line |
| `java.lang.String` | `shortForm()` |
| `boolean` | `wantsValue()`             Tells whether or not this option wants a value |

**Methods inherited from class java.lang.Object**

`clone, equals, finalize, getClass, hashCode, notify, notifyAll, toString, wait, wait, wait`

**Constructor Detail**

### CmdLineParser.Option

```
protected CmdLineParser.Option(java.lang.String longForm,
                               boolean wantsValue)
```

---


### CmdLineParser.Option

```
protected CmdLineParser.Option(char shortForm,
                               java.lang.String longForm,
                               boolean wantsValue)
```


**Method Detail**

### shortForm

```
public java.lang.String shortForm()
```

---


### longForm

```
public java.lang.String longForm()
```

---


### wantsValue

```
public boolean wantsValue()
```

:   Tells whether or not this option wants a value

---


### getValue

```
public final java.lang.Object getValue(java.lang.String arg,
                                       java.util.Locale locale)
                                throws CmdLineParser.IllegalOptionValueException
```

:   **Throws:**: `CmdLineParser.IllegalOptionValueException`

---


### parseValue

```
protected java.lang.Object parseValue(java.lang.String arg,
                                      java.util.Locale locale)
                               throws CmdLineParser.IllegalOptionValueException
```

:   Override to extract and convert an option value passed on the
    command-line

    :   **Throws:**: `CmdLineParser.IllegalOptionValueException`


---


|  |  |  |
| --- | --- | --- |
| **Overview** \| **Package** \| **Class** \| **Use** \| **Tree** \| **Deprecated** \| **Index** \| **Help** | |  |
| **PREV CLASS**   **NEXT CLASS** | **FRAMES**    **NO FRAMES**     **All Classes** |  |
| SUMMARY: NESTED \| FIELD \| CONSTR \| METHOD | DETAIL: FIELD \| CONSTR \| METHOD |  |


---
